# Offsetting Dense Particle Sedimentation in Microfluidic Systems

**DOI:** 10.3390/mi15091063

**Published:** 2024-08-23

**Authors:** Tochukwu Dubem Anyaduba, Jesus Rodriguez-Manzano

**Affiliations:** Department of Infectious Disease, Faculty of Medicine, Imperial College London, London W12 0HS, UK; j.rodriguez-manzano@imperial.ac.uk

**Keywords:** microfluidics, beads, sedimentation, cloud point, droplet microfluidics, phase change, surfactant, hindered settling, Richardson–Zaki, Stokes law, fluid dynamics, fluid splitting, fluid metering, dense particles

## Abstract

Sedimentation is an undesirable phenomenon that complicates the design of microsystems that exploit dense microparticles as delivery tools, especially in biotechnological applications. It often informs the integration of continuous mixing modules, consequently impacting the system footprint, cost, and complexity. The impact of sedimentation is significantly worse in systems designed with the intent of particle metering or binary encapsulation in droplets. Circumventing this problem involves the unsatisfactory adoption of gel microparticles as an alternative. This paper presents two solutions—a hydrodynamic solution that changes the particle sedimentation trajectory relative to a flow-rate dependent resultant force, and induced hindered settling (*i*-HS), which exploits Richardson–Zaki (RZ) corrections of Stokes’ law. The hydrodynamic solution was validated using a multi-well fluidic multiplexing and particle metering manifold. Computational image analysis of multiplex metering efficiency using this method showed an average reduction in well-to-well variation in particle concentration from 45% (Q = 1 mL/min, n = 32 total wells) to 17% (Q = 10 mL/min, n = 48 total wells). By exploiting a physical property (cloud point) of surfactants in the bead suspension in vials, the *i*-HS achieved a 58% reduction in the sedimentation rate. This effect results from the surfactant phase change, which increases the turbidity (transient increase in particle concentration), thereby exploiting the RZ theories. Both methods can be used independently or synergistically to eliminate bead settling in microsystems or to minimize particle sedimentation

## 1. Introduction

Interest in using microparticles as delivery systems in various technologies has been widely researched, especially in combination with microdroplets for biological applications [1,2,3,4,5,6]. This is partly due to the high surface-to-volume ratio and the ease of immobilizing bio-recognition molecules on these materials, as well as the potential for compartmentalized single-molecule assays [7,8]. Unfortunately, challenges with bead settling confound these applications [3,6,9]. Offsetting particle density poses a challenge when loading microparticles into encapsulation devices because the higher-density particles sediment in the fluidic channels, causing a non-homogeneous distribution of microparticles in droplets. One method of resolving this challenge involves suspending the particles in equally dense fluids or introducing humectants such as glycerol [3,10]. However, an adequate amount of the humectants for increased bead buoyancy may be required at concentrations that may be inhibitory to the intended bio-applications, such as nucleic acid amplification technologies [11]. Researchers have also circumvented sedimentation problems by using gel beads [12,13,14,15]. While these have been used successfully in ensuring the binary distribution of beads in droplets without sedimentation issues, their non-Newtonian rheological properties make them difficult to handle. The use of channels with aspect ratios close to the particle diameter is another method for maintaining a single streamline, ensuring that only one particle is queried by the continuous phase at the point of encapsulation. However, considering that these beads are hard-shelled, their packing density may prohibit the possibility of closed packing in narrow channels. Additionally, as sedimentation velocity depends on the mass and size of the particles, the use of smaller particles is also an option; however, this may impact the capacity to carry an adequate amount of biomolecules of interest. Price and Paegel [3] presented a potentially simple solution by exploiting the sedimentation potential of the beads using a hopper system. However, they found that it took 0.8 h (17 µm TetanGel resin beads) and 3.8 h (2.8 µm magnetic beads) to introduce the beads before single-bead encapsulation. Kim et al. [2] successfully developed a pneumatic system that was capable of trapping and releasing beads, thus creating a deterministic encapsulation of a defined number of beads per droplet. This system, however, involves complex implementations of pumps and valves, thus making it unfit for low-cost and low-complexity applications. Mechanical agitation has also been successfully adopted; however, this complicates the system and could make integration into a unified product difficult. Applications requiring equal spatial distribution of particles are also impacted by sedimentation, which is compounded by non-slip conditions in laminar flow between parallel plates. For particles in such systems, wall lift and drag forces have been shown to depend on shear rate, especially at very low Reynold’s numbers [16]. In this paper, simplistic solutions to sedimentation, which can be applied to most particle-based systems, are exemplified in two different forms. A flow-rate-dependent method that alters the sedimentation trajectory of suspended particles was applied to a microfluidic particle metering system while induced hindered settling was applied to particles in suspension.

## 2. Materials and Methods

### 2.1. Design and Fabrication of Fluid-Metering Chip

**Figure 1:**
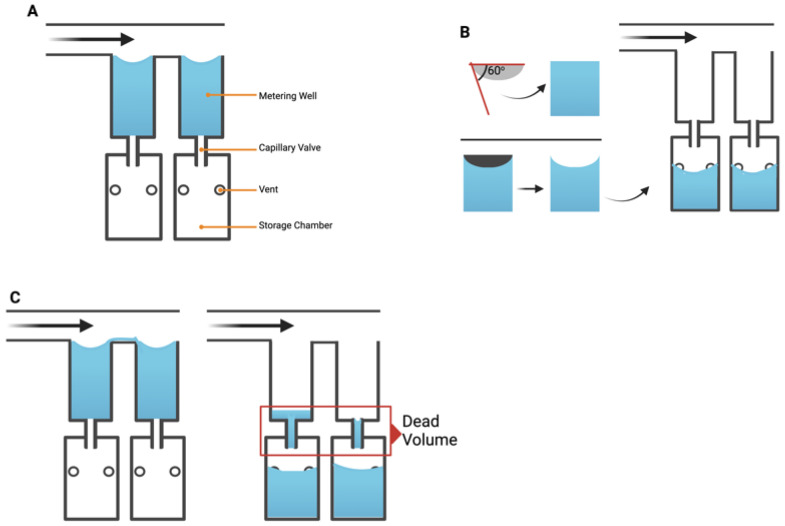
Minimal illustrations of the chip sub-units showing critical design elements. (**A**) Metering and storage chambers separated by a capillary valve (**B**) Illustration showing method used to determine the position of the venting holes in the bottom (storage) chamber (**C**) Once fluid in the storage chamber wicks through the venting holes to a PTFE filter, the pressure within the chamber increases above the Laplace pressure. this prevents further filling of the storage chamber.

The chip was designed as a 16-well manifold for fluid and particle metering devices in which the metering chambers are separated from a lower storage chamber by a capillary valve (Figure 1A). Having both chambers was necessary to prevent one of the consequences of manifold systems, which is sequential filling. This would entail that each well will be filled to the brim before the next, thereby leaving no headspace to allow for further fluid manipulations such as mixing. The lower storage chambers were perforated at positions modeled to provide a convex meniscus at the approximate intended fill volume. To achieve this on a 3D plane, a 60° hemisphere mimicking the hydrophilic contact angle between the chip surface and the buffer was used to cut an extrusion of the 3D-model infill until the desired fill volume was achieved on the model (Figure 1B). During assembly, the perforations were plugged with polytetrafluoroethylene (PTFE) membranes such that wicking of the metered volume into the membranes triggered an increase in the chamber pressure, thereby preventing further emptying of the top metering chamber (Figure 1C). All 3D models were designed using SolidWorks (Dassault Systèmes) and printed using ProFLuidics 285D digital lightprocessing (DLP) 3D printer (CADworks3D). The design files are included as Supplementary File (SF1).

**Figure 2:**
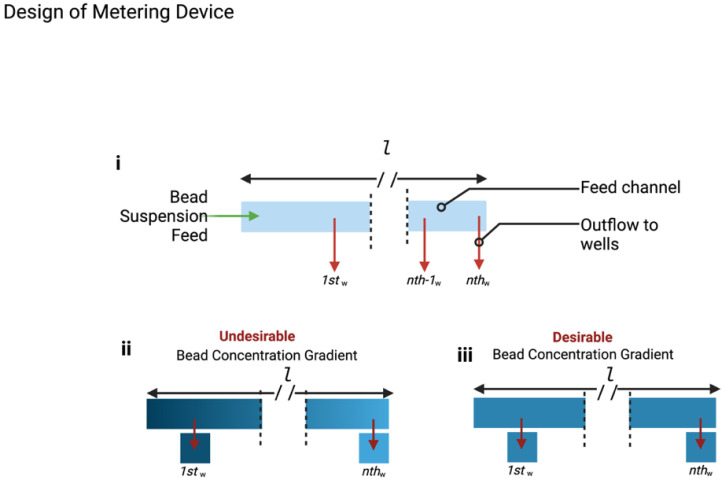
(**i**) Illustration of the fluidic metering device showing the shared feed channel and the tributary wells (outflow channels). (**ii**) Illustration of undesirable (**iii**) desirable effect of bead settling as a result of sedimentation across the feed channel of length, *l*.

As illustrated in Figure 2, a consequence of the increased number of metering wells, n, is an increase in length of the flow path, *l* (Figure 2i), which consequently leads to a particle concentration gradient, in which the first well (Figure 2ii) contains a significantly greater concentration of suspended particles than the nth well due to bead settling. This challenge necessitates the need for a mechanism of counteracting or reducing the sedimentation of the beads to improve metering efficiency (Figure 2ii,iii).

### 2.2. Hydrodynamic Interruption of Sedimentation

The interaction of particles of different sizes and shapes have been the subject of plenty of research, especially in environmental studies. However, there is a dearth of empirical demonstration of hydrodynamic interruption of particle sedimentation, especially in microfluidic systems. Here, the fabricated fluidic and particle-metering chips were used to demonstrate this phenomenon. The fabricated chips were connected to a syringe pump (Legato, KD Scientific, USA) via a modified Eppendorf tube, which held the bead suspension. For each experimental run, the syringe pump was programmed to run at a defined, arbitrarily chosen volumetric rate, Q = 1, 3.5, 5, and 10 mL/min until the feed channel was completely emptied. Each experimental condition was replicated (for Q = 1 mL/min, n = 2; for Q = 3.5, 5 and 10 mL/min, n = 3) to determine the reproducibility of the results. Before each run, the bead vial was vortexed to ensure uniform bead distribution, then connected to the metering device and pumped within 10 s, thereby preventing pre-settling of the beads. Visual data were collected using M50 Mark II mirrorless camera (Canon, USA) at 60 frames/s for computational image analysis via the algorithm described in the data acquisition and analysis section.

### 2.3. Induced Hindered Settling (i-HS)

The Richardson–Zaki modification of Stokes’ law [17,18] (Equation (1)) shows that at higher particle concentrations, the particle settling velocity decreases due to particle–particle interactions. This principle suggests that if we could increase the particle concentration in the buffer, we could delay or overcome particle settling. For biomedical applications, however, the particle concentrations are predetermined empirically for optimal performance and cost reasons and, as such, cannot be indefinitely increased to exploit the Richardson–Zaki principle. This principle could be exploited only if there was a way to transiently increase the particle concentration (or increase the turbidity of the particle-suspending solution) without introducing additional particles. This is the basis of the *i*-HS alternative.

(1) $$\frac{V_{t}}{V_{0}}={\left(1-⌀\right)}^{n}$$

where $V_{t}$ = settling velocity in turbid fluid,

$V_{0}$ = settling velocity in clear fluid,
*n* = exponent of reduction in settling velocity,
*⌀* = volume fraction of suspended particles in the fluid (turbidity)

**Figure 3:**
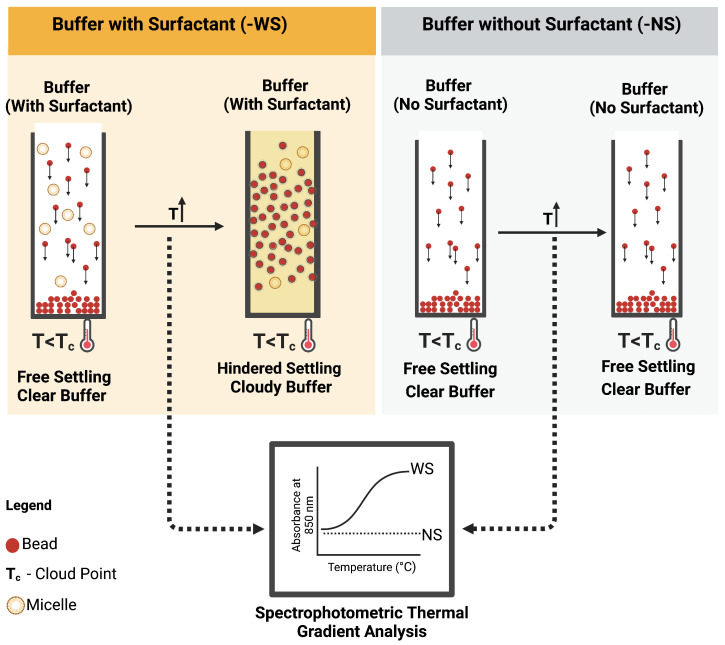
Schematic representation of induced hindered settling and spectrophotometric thermal gradient analytical protocol.

To ascertain the feasibility of the *i*-HS, the cloud point of the surfactant in the bead suspension buffer was exploited. Cloud point refers to a physical property (temperature) of non-ionic surfactants at which their dissolution in liquid reverses, leading to liquid–liquid phase separation. At this temperature, as illustrated in Figure 3, the surfactants form micelles, thereby increasing the turbidity (cloudiness) of the suspending solution. These micelles transiently act as particles, thereby allowing the empirical exploitation of the Richard–Zaki theory. As a preliminary analysis of this concept, the buffer was modified with and without ECOSURF EH-9 and designated with the prefixes -WS and -NS, respectively, and analysed via a spectrophotometric thermal gradient (STG) using BioTek Epoch 2 microplate spectrophotometer (Agilent, USA) in a 96-well plate. The choice of the non-ionic surfactant, ECOSURF EH-9, was informed by its biodegradability, among other functions such as wetting. Hypothetically, an increase in the turbidity of the -WS buffer relative to the turbidity of the -NS buffer would signal the feasibility of the concept.

Subsequently, bead suspensions of the same concentration as in previous experiments were added to clear glass vials and grouped according to the experimental protocol This setup enabled the investigation of the effect of the surfactant cloud point on bead settling. Test temperatures that can be tolerated by downstream processes (50 ± 10 °C), and at which cloud point was induced were chosen from different temperature points on the STG data for further sedimentation analyses. Raw STG data are included as Supplemetary Data, SD1.

### 2.4. Data Acquisition and Analysis

Replicate (n = 12) turbidimetric measurements at different temperatures and data collection were achieved using a spectrophotometer at a near-infrared (NIR) wavelength of 850 nm. For bead settling experiments, heating of the glass vials for bead settling was performed using a Benchmark Multitherm shaker and cooling device. Visual data in the form of video recordings were collected for the assessment of bead settling and suspension homogeneity in the vials using M50 Mark II Mirrorless camera (Canon, USA) at 60 frames per second). Each test was run in duplicates.

**Figure 4:**
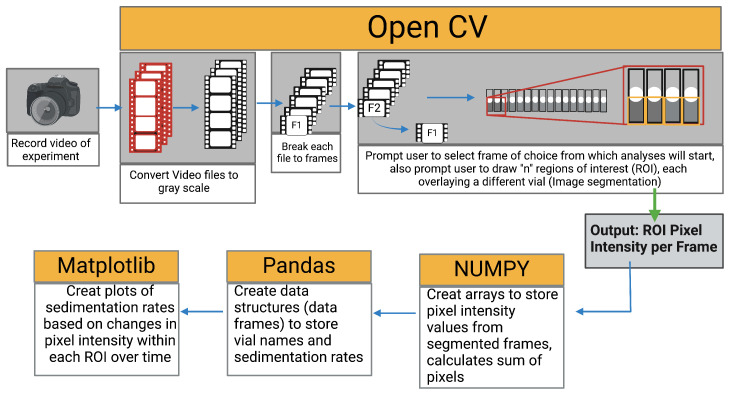
Still and a video image processing algorithm for feature extraction and numerical data retrieval.

The collected videos were analysed using Python 3.6 computer vision library (OpenCV) following the algorithm shown in Figure 4. To determine the bead settling velocity in the vials, the intensity of the pixels within the region of interest (region covered by the bead suspension) was monitored throughout the video timeline. By monitoring the pixel intensity over time, the presence and later absence of beads within a pixel signals the settling of the beads. The sedimentation rate was determined as the rate of change of gray value (Equation (2)).

(2) $$\frac{\partial G}{\partial t}=-V_{s}$$

where $\frac{\partial G}{\partial t}$ = rate of change of gray values over time, $V_{s}$ = sedimentation rate.

The pixel intensity (mean gray value) refers to the brightness of a pixel in an image. From the grayscale images, the intensity was measured on a scale of 0–255, where 0 represents black and 255 represents white. The mean gray values correspond to the concentration of the beads or the homogeneity of the bead suspension.

## 3. Results

### 3.1. Fabrication of Particle Metering Chip

To prevent premature emptying of the top metering wells, the dimensions of the feed channel were modeled using 2023 SolidWorks Flow Simulations software (Dassault Systèmes). The capillary stop valves were designed to have a Laplace pressure of 403 Pa, calculated using Equation (3).

(3) $$\Delta P=2*\gamma \left(\frac{1}{w}+\frac{1}{h}\right)\cos\theta_{c}$$

where $\theta_{c}$ = fluid contact angle = 2, *w* = valvel width = 0.5 mm, *h* = valve height = 1.25 mm, $\gamma =\gamma_{\mathrm{water}}$ = surface tension = 0.072 N/m.

After printing, the geometric conformance of the 3D-printed capillary valves to the CAD model was determined using optical metrology (Keyence). This was determined to be 0.49 ± 0.02 mm (n = 10), thus conforming with the design parameters (Equation (3)).

**Figure 5:**
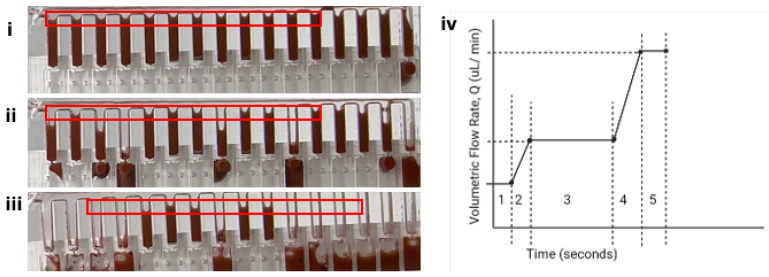
(**i**–**iii**) shows the effect of asynchronous emptying of the metering wells. (**iv**) shows the pump ramp program: (**2**) Ramp from 0.1 mL/min to 1 mL/min in 5 s. (**3**) Hold at 1 mL/min for 72 s (*72 s is the time required to completely empty the surrogate elution chamber per my setup + time to empty the feed well*) (**4**) Ramp from 1 mL/min to 10 mL/min in 5 s (*5 s–arbitrarily chosen—being careful to ensure the syringe pump could handle that ramp)* (**5**) Hold at 10 mL/min for 6 s (10 mL/min for 6 s ensures 690 µL is pushed from the all metering wells while keeping the unit pressurized). The positive pressure is maintained momentarily to ensure all wells are emptied simultaneously.

A major consequence of manifold splitting results from the interaction between the buffer, the shared walls between wells, the material surface properties, and the flow regime (Figure 5iii). This interaction led to an undesired siphon effect, whereby asynchronously emptied wells drew fluid from neighboring wells, leading to metering inefficiencies. This was resolved by reprogramming the flow regime to ensure synchronous emptying of the wells via an instantaneous increase in Laplace pressure (Figure 5iv).

### 3.2. Hydrodynamic Interruption of Sedimentation

**Figure 6:**
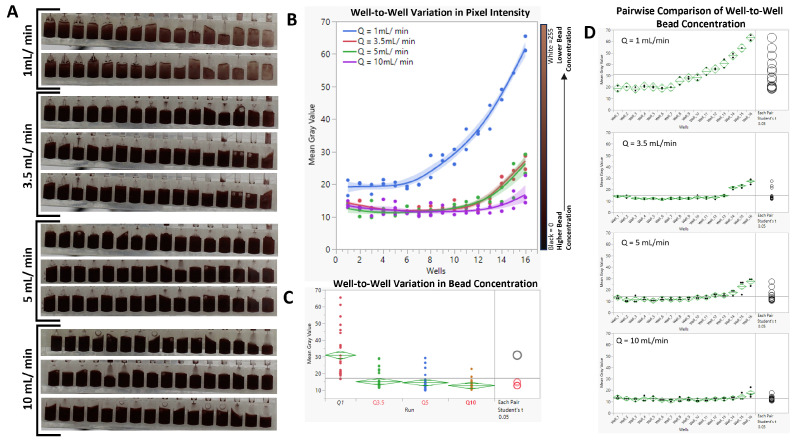
(**A**). Cropped frames showing well-to-well variation in bead concentration per flow rate. (**B**). Distribution pattern of the suspended beads in response to changes in the volumetric flow rate. (**C**). Comparison of the effect of flow rate on the mean bead concentration. (**D**). Pairwise comparison of well-to-well bead concentrations.

As shown in Figure 6A,B, at a volumetric flow rate of Q = 1 mL/min, much of the beads eluted early in the experimental run, forcing the mean gray value (MGV) of wells 1 through 7 to be significantly lower (higher bead concentration) than that of wells 8–16 (Figure 6D). Pairwise comparisons (Student’s *t* test, alpha = 0.05) of the MGVs of the 16 wells from Q = 1 mL/min to the other tested Qs revealed a significant difference between them, with *p* values < 0.0001 (Figure 6C). While increasing Q from 3.5 mL/min to 10 mL/min did not significantly affect the average concentration of the beads (*p* values: Q3.5 − Q10 = 0.107; Q5 − Q10 = 0.231; Q3.5 − Q5 = 0.675), analyses of the data from each flow rate showed improved bead distribution (Figure 6D). For Q = 3.5 mL, wells 14–16 had significantly lower bead concentrations; for Q = 5 mL/min, wells 15 and 16 had significantly lower bead concentrations; and for Q = 10 mL/min, only well 16 had a significantly lower bead concentration. Video recording of the analyses can be found in the Supplementary Folder, SD2.

### 3.3. Sedimentation Offset via Induced Hindered Settling

**Figure 7:**
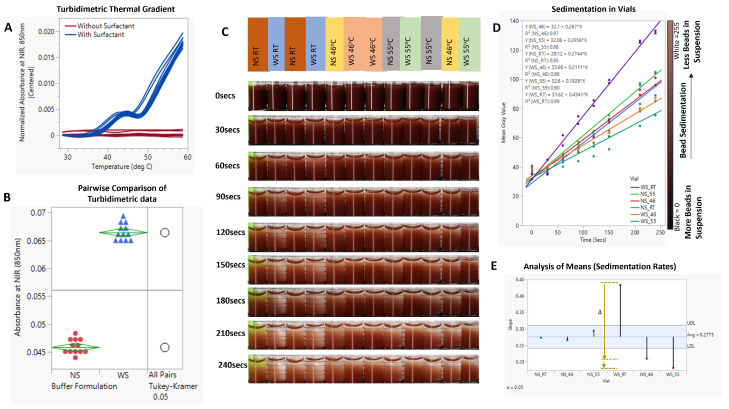
(**A**). Spectrophotometric thermal gradient (STG) analysis of buffers with (-WS) and without (-NS) surfactants. (**B**). Pairwise comparison of STG results. (**C**). Timelapse images showing bead sedimentation in glass vials. (**D**). XY plots of different experimental conditions showing the temporal rate of change in pixel intensity. (**E**). Analysis of means (ANOM) of the sedimentation gradients (α = 0.05). Video data of the experimental setup is included in Supplementary Folder, SD3.

Phase separation in the particle suspension buffer was induced and confirmed via spectrophotometric analyses, which showed a temperature-driven increase in turbidity (Figure 7A). As this change is reversible [19], it offers a perfect solution for increasing the probability of particle–particle interactions and, consequently, hindering settling. Incubation of the WS buffer at 55 °C resulted in a ~45% increase in turbidity (Figure 7B).

As shown in Figure 7C,D, this phenomenon translated to a reduction in bead settling velocity. In accordance with the theory of hindered settling, an increase in the bead suspension buffer temperature and, consequently, the cloud point of the surfactant reduced the sedimentation rate of the beads by 58% (Figure 7D,E). Continuous or controlled heating of the beads in a vial showed a similar result of improved particle metering. Notably, the presence of surfactants, while necessary for fluid flow, increases the bead settling velocity, as shown in Figure 7C–E—WS_RT (slope = 0.4341) vs. NS_RT (slope = 0.2744). As shown in Figure 7E, there was no significant difference in sedimentation rate between -NS buffer at room temperature and one heated to 46 °C (NS_46 °C vs. NS_RT); however, increasing the temperature of the -NS group to 55 °C led to an increase in the rate of sedimentation (NS_46—slope: 0.267, NS_55—slope: 0.296).

## 4. Discussion

### 4.1. Hydrodynamic Interruption of Particle Sedimentation

Particle sedimentation occurs because of the action of gravitational pull on the particles. The rate at which particles sediment, $V_{s},$ is influenced by factors such as particle volume and the density of the suspending fluid.

(4) $$V_{s}=g\frac{{2r}^{2}}{{9\eta }_{0}}\left(\frac{m_{p}}{v_{p}}-\;\rho_{f}\right)$$

$V_{s}=\;$ Sedimentation velocity at infinite dilution, *r* = particle radius, $\eta_{0}$ = viscosity of suspending fluid, $m_{p}\;$= mass of particle, $v_{p}$ = volume of particle, $\rho_{f}$ = density of suspending fluid, g = |g| = 9.8${\;m/s}^{2}$.

These, in turn, also influence the drag force, ($F_{d}$), which in simple terms, the drag force refers to the resistance force exerted by a fluid to the downward motion of the particles.

(5) $$F_{d}=\;\frac{1}{2}{[\rho }_{f}C_{d}A{V_{s}}^{2}]$$

where $F_{d}$ = drag force, *V* = particle velocity, *A* = particle area, $C_{d}$ = drag coefficient = 24Re for spheres [20], therefore:

(6) Fd=12[ρfAVs2Re]

Substituting $g\frac{2r^{2}}{9\eta_{0}}\left(\frac{m_{p}}{v_{p}}-\rho_{f}\right)$ for $V_{s}$ in Equation (5)

(7) Fd=12[ρfA[g2r29η0(mpvp−ρf)]2Re]WhereRe=Reynold’snumber

**Figure 8:**
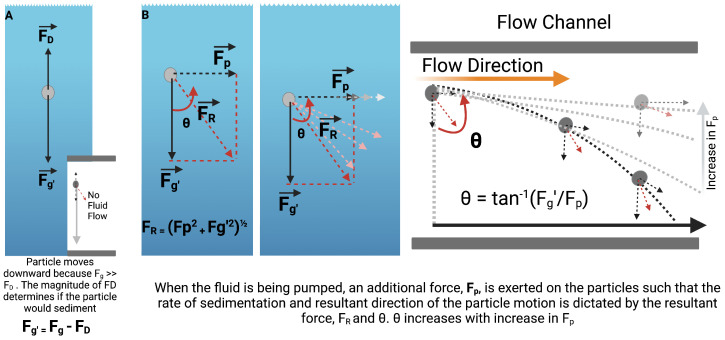
Working theory for hydrodynamic interruption of particle sedimentation. (**A**) At steady state, particles in suspension are acted on by two forces F_D (drag force) and F_g (gravitational pull) whose resultant is denoted by F_g’ and is determined by the magnitude of the counteracting forces. (**B**) Shows the resultant of forces acting on particles suspended in fluids in flow.

As shown in Equation (7), several factors contribute to the drag force exerted on the particles. Consider particles at a steady state, as illustrated in Figure 8, the particles sediment in response to the resultant of opposing forces, drag force, and gravity.

(8) $$F_{g^{\prime }}=\;F_{g}-\;F_{d}$$

However, in a fluidic state, the rate and direction of sedimentation is largely determined by the magnitude of the force, $F_{p}$ applied to the particle due to pumping.

As the deviation of the particle from a straight downward sedimentation is given by:

(9) $$\theta =\;\tan^{-1}\left(\frac{F_{g^{\prime }}}{F_{p}}\right)$$

Theoretically, at constant |g’|, increasing $F_{p}$ would result in a concomitant increase in θ towards 90°.

(10) $$\lim_{F_{p}\to \infty }\theta (F_{p})=90^{\circ }$$

(11) $$\int_{F_{p}}^{\infty }\frac{d\theta }{dF_{p}}\;dF_{p}=90^{\circ }-\theta \left(F_{p}\right)$$

Considering this theory, as $F_{p}$ = ma, where m=mass; and a = acceleration = $\frac{\mathrm{Fluid}\;\mathrm{Velocity}}{\mathrm{time}}$, $F_{p}$ can be increased via the volumetric flow rate.

Empirical findings from the data above validate the working theory as stated in Equations (9)–(11); thus, as the acceleration of the beads in solution increase as a result of the increase in Q of the suspending fluid, gravitational pull on the particles is counteracted. The particles therefore remain homogenized in solution long enough to be evenly distributed. This method, although simplistic and easy to implement in most systems, may be impractical for shear-averse processes where high flow rates (deviation from laminar flow) could impact the structural integrity of the suspended particles. Additionally, as shown in Figure 6C, increasing Q beyond a certain threshold yields very minimal improvement, which may not justify the high volumetric flow rate requirement. These limitations, therefore, necessitate the need for a more shear-tolerant alternative that would not only alter the sedimentation trajectory, but also significantly reduce it.

### 4.2. Induced Hindered Settling

Although ECOSURF EH-9 has a cloud point of 64 °C at 10 wt% actives aqueous solution, the spectrophotometric thermal gradient data (Figure 7A) show that at 0.1 wt%, turbidimetric changes could be initiated at temperatures lower than the cloud point. This finding improves on the existing knowledge that heating solutions containing non-ionic surfactants to temperatures above the surfactant cloud point (T_c_) could induce a phase change [21]. While the exact role of the salts in the proprietary buffer on the lower cloud point was not explored, data from [22] suggest that the cloud point of non-ionic surfactants could be lowered via the addition of salts. Figure 7A also shows a biphasic change in buffer turbidity relative to temperature increase. The primary reason for this biphasic relationship could not be determined from secondary data; however, a thermally-induced phase change due to surfactant cloud point could explain an increase in turbidity. This phase change results in the formation of surfactant micelles that interact with the particles, thereby conforming to the Richardson–Zaki theory. Although this phase change can also be induced chemically via the addition of ionic surfactant and electrolytes, for this application, micellar aggregation occurred because of thermally induced reduction in the hydration of oxyethylene oxygen in hydrophilic groups, which leads to micellar aggregation. This phenomenon has been widely adopted in environmental studies and in separation science as a form of cloud point extraction (CPE) [21,23,24,25,26]. While encouraging, most applications requiring microparticle manipulation may not be tolerant to heating. For such applications, the choice of surfactants with cloud points closer to tolerable temperatures or chemical tuning [22] of the surfactant cloud point may be ideal.

## 5. Conclusions

Various biomedical applications, especially in microfluidic systems, require microparticle handling [27]. The development of specialized devices for continuous mixing [28,29,30,31] or a complete switch to gel beads has been mostly adopted due to the challenge of bead settling. However, these factors increase the cost and complexity of the system. This paper presents two simplistic solutions, hydrodynamic and i-HS solutions. Both solutions exploit rudimentary components in biomedical platforms, thereby not adding to the cost. While they can be applied independently, they can also be combined to achieve a uniform distribution of particles of various sizes or to lower their settling velocity for fluidic applications. This was successfully applied in particle metering and distribution manifold. A limitation of both systems is the requirement for an initial homogenization step and continued heating for the i-HS (optional). Moreso, the thermal induction of hindered settling as demonstrated in this research may not be ideal for most biomedical systems. However, thermal i-HS may not be necessary, considering the possibility of non-thermal cloud point tuning [22]. These principles can be adopted in particle-laden flows involving the need for mechanistic encapsulation of microparticles. More so, to the best of our knowledge, there are no published studies that explore the integration of mechanical agitation and multi-well particle metering to set a comparative baseline. This study establishes such a baseline. Further, continuous mechanical agitation of particle suspensions upstream does not influence particle behaviours downstream where convective flows are negligible; as such, regardless of mechanical mixing in bulk solution, additional measures are required to forestall undesirable sedimentation of particles.

## Data Availability

Data for this article, including supplementary pictures, videos, Python codes, and raw data files, are available in the Supplementary Material.

## Supplementary Materials

The following supporting information can be downloaded at: https://doi.org/10.5281/zenodo.13309460 (accessed on 19 August 2024).
